# A New Look at the Structures of Old Sepsis Actors by Exploratory Data Analysis Tools

**DOI:** 10.3390/antibiotics8040225

**Published:** 2019-11-14

**Authors:** Antonio Gnoni, Emanuele De Nitto, Salvatore Scacco, Luigi Santacroce, Luigi Leonardo Palese

**Affiliations:** 1 SMBNOS—Università degli Studi di Bari, 70124 Bari, Italy; antonio.gnoni@uniba.it (A.G.); emanuele.denitto@uniba.it (E.D.N.); salvatore.scacco@uniba.it (S.S.); 2 Ionian Department, Microbiology and Virology Lab Unit, University Hospital of Bari, Università degli Studi di Bari, 70124 Bari, Italy

**Keywords:** Sepsis, allosteric, albumin, cyclooxygenase, hemoglobin, PCA, random projection, pathophisiology, bioinformatics tools, clinical chemistry

## Abstract

Sepsis is a life-threatening condition that accounts for numerous deaths worldwide, usually complications of common community infections (i.e., pneumonia, etc), or infections acquired during the hospital stay. Sepsis and septic shock, its most severe evolution, involve the whole organism, recruiting and producing a lot of molecules, mostly proteins. Proteins are dynamic entities, and a large number of techniques and studies have been devoted to elucidating the relationship between the conformations adopted by proteins and what is their function. Although molecular dynamics has a key role in understanding these relationships, the number of protein structures available in the databases is so high that it is currently possible to build data sets obtained from experimentally determined structures. Techniques for dimensionality reduction and clustering can be applied in exploratory data analysis in order to obtain information on the function of these molecules, and this may be very useful in immunology to better understand the structure-activity relationship of the numerous proteins involved in host defense, moreover in septic patients. The large number of degrees of freedom that characterize the biomolecules requires special techniques which are able to analyze this kind of data sets (with a small number of entries respect to the number of degrees of freedom). In this work we analyzed the ability of two different types of algorithms to provide information on the structures present in three data sets built using the experimental structures of allosteric proteins involved in sepsis. The results obtained by means of a principal component analysis algorithm and those obtained by a random projection algorithm are largely comparable, proving the effectiveness of random projection methods in structural bioinformatics. The usefulness of random projection in exploratory data analysis is discussed, including validation of the obtained clusters. We have chosen these proteins because of their involvement in sepsis and septic shock, aimed to highlight the potentiality of bioinformatics to point out new diagnostic and prognostic tools for the patients.

## 1. Introduction

Sepsis is a life-threatening condition that accounts for numerous deaths worldwide, usually as complications of community infections (i.e., pneumonia, etc.), or infections in hospitalized patients. Sepsis and septic shock, its most severe evolution, involve the whole organism, recruiting and producing a lot of molecules, mostly proteins. Protein functions are closely related with their structure, and the discovery of meaningful structure-function relationships is of overwhelming importance in biochemistry. Conformational changes in proteins have been known for a long time and are crucial for the biological activity of these molecules. These changes range from subtle side-chain displacement or change in the flexibility in some loop to large whole domain motions. Conformational changes have been involved in the enzymatic activities of proteins, in the recognition of substrates and in the protein-protein interactions. Because of their importance, numerous experimental and computational techniques were developed to allow the extensive characterization of these conformational changes so that it is virtually impossible to remember them all here. In recent years there has been a considerable increase in the ability to produce high-quality three-dimensional structures of proteins. To date more than 126,000 structure are in the Protein Data Bank (PDB) [1,2,3]. This number continues to grow dramatically and for many proteins multiple entries are present in the PDB. Important information about conformational states of specific proteins can be extracted by the analysis of these redundant entries for the same protein, generally obtained in different conditions. For single protein, redundant data sets can be analysed using various mathematical tool [4]. A classical approach is the principal component analysis (PCA), a multivariate statistical method based on the covariance of data [5,6,7]. This method has a wide range of applications in today’s data science [8,9,10,11]. If the number of data (or different structures in the case of proteins) is sufficiently high, PCA even makes it possible to reconstruct the main modes of protein motion starting from the (static) crystallographic structures, in excellent agreement with the experimental and molecular dynamics data [12,13]. However, the fact that the number of entries in these structural data sets is large but in general not comparable (i.e., less than) the number of degrees of freedom that are needed to describe a typical protein imposes several constraint to the algorithm to be used in such analyses. In this case, to perform a PCA type analysis, it is necessary to use specialized state of the art algorithms [14], which are also able in this type of data sets to reveal information on the dynamics [15] or the presence of functionally important clusters [16].

The reader should consider that crystal structures represent time and space averages of all molecules present within the crystal lattice (which is not perfect). Conformational variations can provide information about the flexibility or movement of regions of protein structure that might be important for function and ligand binding. Even in the case of a single structure corresponding to the minimum of a potential well, the protein is actually a family of structures that can be explored as a result of thermal motion. Particularly in the case of subtle structural differences it is necessary to consider not only if and how important are these, or are they related to some functional aspect of the protein, but first of all if they can be simply due to thermal motion (so to speak, frozen in the coordinates provided in the PDB), or also to refinement errors [17].

Here we show that a simple algorithm based on random projection [18] performs well in the dimensionality reduction and unsupervised clustering of protein structure data sets. Furthermore, if data clusters are effectively well separated, this will be true even in the case of random projection. Therefore, if we find clusters of data in two-dimensional projections obtained by PCA that are not observable even in the random projection, it is possible that the clusters are not reliable. In this case caution is required in the interpretation of the data, which must be integrated with the biochemical knowledge available on the particular proteins. We apply this algorithm in the exploratory data analysis on three model proteins that represent different types of allostery from a structural point of view: a monomeric allosteric protein that exhibits evident structural changes, a case of allostery without dramatic structural changes, and a classical multimeric allosteric protein. All these proteins are involved in various ways in sepsis and, to better understand this process, the study of the conformational changes of existing and newly produced proteins that occur during an infectious process is really interesting.

## 2. Results

### 2.1. The Human Serum Albumin: Allostery in a Monomer

The human serum albumin (HSA) [19], the most abundant protein in plasma, is a monomeric multi-domain molecule. HSA is a non-glycosylated, all-α protein chain of 65 kDa, with a globular heart-shaped conformation containing three homologous domains. Each domain is composed by two subdomains. It is an important transport protein with different binding sites able to accommodate a number of chemically different ligands. HSA represents the main carrier for fatty acids, for which there are seven binding sites. It is also a depot and carrier for exogenous compounds (mainly, but not exclusively at the so called Sudlow’s sites I and II), thus affecting the pharmacokinetics of many drugs. Hypoalbuminaemia is often associated with sepsis and/or critical illness, and the supplementation of HSA still remains controversial in these patients [20]. In fact, the function of HSA is fundamental in the infective and septic process, and is closely related to specific conformational modifications, influencing the whole health status of the patients [21]. It is worth noting that a large number of structural and functional works on HSA have lead to the conclusion that two structures, possibly related to the presence of fatty acids, are discernible for this protein [19,22]. Short chain fatty acids (SCFAs), a common product of microbial metabolism, affect albumin production and metabolism, so they have a role in the evolution of the septic patients [23]. In fact, they directly influence the hepatic albumin metabolism [24]. This three-domain organization of HSA is at the root not only of its extraordinary ligand binding capacity, but also of the allosteric control of this last. The HSA structure and reactivity (and also its enzymatic activity) is affected reversibly by pH and ligands, such as fatty acids, heme or drugs.

**Figure 1:**
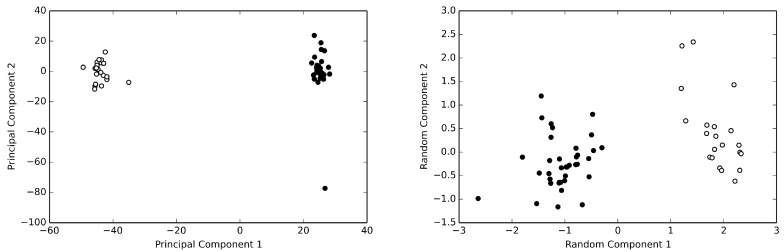
The human serum albumin data set. Principal component analysis (**left panel**) and random projection analysis (**right panel**) of the of the normalized HSA data set are reported. HSA structures without bound fatty acids are reported as withe circles, while those with bound fatty acids are reported as black circles. Both methods clearly allow to recognize two clusters of structures. Note the different level of dispersion provided by the two methods.

Among the available structures, we selected 58 structure for the analysis. This data set has been described in details elsewhere [18]. The α-carbon atom Cartesian coordinates of HSA were extracted and arranged in a data matrix, such that each row represented a single HSA structure. Thus, the data matrix was composed of 58 rows and 1695 columns (565 α-carbon atoms were finally included in the analysis [18]). This is a degenerated data set, as it is impossible to obtain the true correlation matrix of a multivariate system with 1695 degree of freedom by using only 58 samples. As recalled above, in order to reduce the dimensionality and to obtain an unsupervised clustering of the structures present in the data set, it is possible to use algorithms that estimate the principal components. Using the truncated singular value decomposition (SVD) algorithm [14] to estimate the principal components, two clusters of structures for the HSA data set can be discerned, as can be seen from Figure 1. However, the same clusters can be obtained by the simple random projection algorithm. As can be easily appreciated by inspecting the figure, these analyses clearly demonstrate that the only discriminant for such a structural switch in the whole data set is the presence or absence of bound fatty acids [18].

### 2.2. The Cyclooxygenase: Allostery without Conformational Change

The cyclooxygenase (COX), also known as prostaglandin H${}_{2}$ (PGH${}_{2}$) synthase or prostaglandin endoperoxide H${}_{2}$ synthase (PGHS), is a membrane bound, heme-dependent bis-oxygenase and hydroperoxidase [25,26,27]. This enzyme participates to the prostanoid synthesis by two sequential reactions: the bis-oxygenation of arachidonic acid (the cycloxigenase reaction) and the reduction of prostaglandin G2 (PGG${}_{2}$) (in the peroxidase site) to form PGH${}_{2}$. In mammals, arachidonic acid is the major prostanoid precursor, which are a subclass of the eicosanoids. COX has a pivotal role in the production of a large number of immune and inflammatory mediators, and the effectiveness of COX inhibition as a treatment for severe sepsis has been extensively studied [28]. Two isoforms of COX can be found in mammals, the constitutive COX-1 and the inducible COX-2. These two isoforms are significantly different in their expression profiles and physiological roles and are involved in various pathological situations. From a structural point of view, and as expected considering the sequence similarity, the two isoforms are quite similar. COX functions as homodimer, and each monomer consists of three domains [26]: an EGF domain at the N-terminal, a membrane-binding domain and a large globular C-terminal domain. This last domain contains the heme binding site and is the responsible of the catalytic activities of these enzymes. The EGF domain participates to the dimer interface and probably to the interaction with membranes. The membrane-binding domain consists of four short amphipathic α-helices. The bulk of COX is represented by the catalytic domain, which is composed essentially by α-helices. Nonsteroidal anti-inflammatory drugs (NSAIDs) are a drug class that inhibit the COX activity. NSAIDs can be divided in two classes: the classical isoform non-specific, that inhibit both COX-1 and COX-2, and the COX-2 inhibitors show a high selectivity for this particular isoform. A large number of studies has demonstrated that COX is a dynamic and flexible molecule that does undergo conformational changes upon binding of heme, substrates and drugs [26].

The fact that COX works as a homodimer and a series of data on its enzymatic activity strongly suggest that this enzyme can undergo to allosteric regulation by its substrates [29,30,31]. However, despite a growing number of crystal structures available in different conditions, no evident ligand-induced conformational changes can be noticed [26]. We have analysed data sets of these enzymes as example of proteins where only tiny (if any) structural changes can be observed. We selected 38 *Ovis ares* COX-1 structures and from these we obtained a 38×1653 matrix representing the Cartesian coordinates of the α-carbon atoms (551 α-carbon atoms). The COX-2 data set included 78 entries from the *Mus musculus* specie, arranged in a matrix of dimension 78×1608 (536 α-carbon atoms).

**Figure 2:**
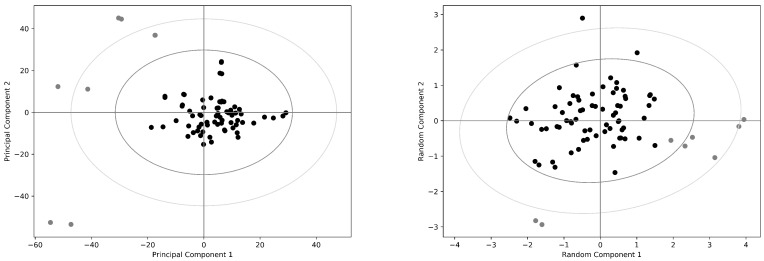
The *Mus musculus* COX-2 data set. Principal component analysis (**left panel**) and random projection analysis (**right panel**) are reported of the normalized data set containing the COX-2 monomers. Outliers in the principal component analysis are reported as gray circles in both panels; 95% and 99% confidence levels are drawn in dark and light gray, respectively. Note that both methods do not identify clearly separate clusters of structures.

The results of the PCA (by the truncated SVD methods recalled above) and the random projection analysis for the COX-2 data set are reported in Figure 2. Both methods show that all the analyzed structures are distributed in a single cluster, in agreement with what is known about the structural variability of this enzyme in different conditions. PCA analysis detects some putative outliers, indicated as gray circles in the Figure, which are located at the peripheral region of the cluster obtained by the random projection algorithm, but not linearly separable from the bulk of structures. Moreover, the outliers distribution is not exactly the same using the two methods: this suggests that, in this case, the separation obtained by PCA is probably strongly influenced by the noise due to the low number of available samples. It should be noted that no meaningful partition of these data can be obtained considering the presence (or absence) of ligands, such as NSAIDs, fatty acids or heme, in agreement with the conclusion that probably only one cluster of structures is actually present in the data set.

**Figure 3:**
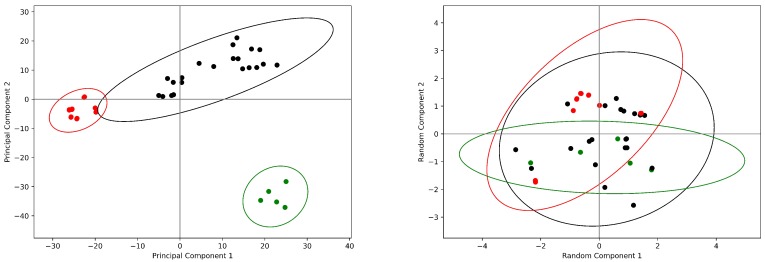
The *Ovis aries* COX-1 data set. Principal component analysis (**left panel**) and random projection analysis (**right panel**) are reported of the normalized data set containing the COX-1 monomers. Principal component analysis identifies on this data set three clusters of structures, indicated as black, red and withe circles. On the contrary the random projection method returns for this data set a single cluster; the entries are colored as in the left panel. The 99% confidence levels for the clusters are reported are drawn in the same color.

The *Ovis ares* COX-1 data set shows different results, depending on the algorithm used for the dimensionality reduction. As indicated by Figure 3, the PCA algorithm describes three different clusters of structures (labeled as red, black and green circles in the Figure). One of this, the one shown in green in the Figure 3, is particularly interesting because it is composed by entries that have been crystallized as monomers with bound fatty acids [32,33,34,35]. The other two clusters are both composed by unliganded molecules or structures containing bound NSAIDs.

**Figure 4:**
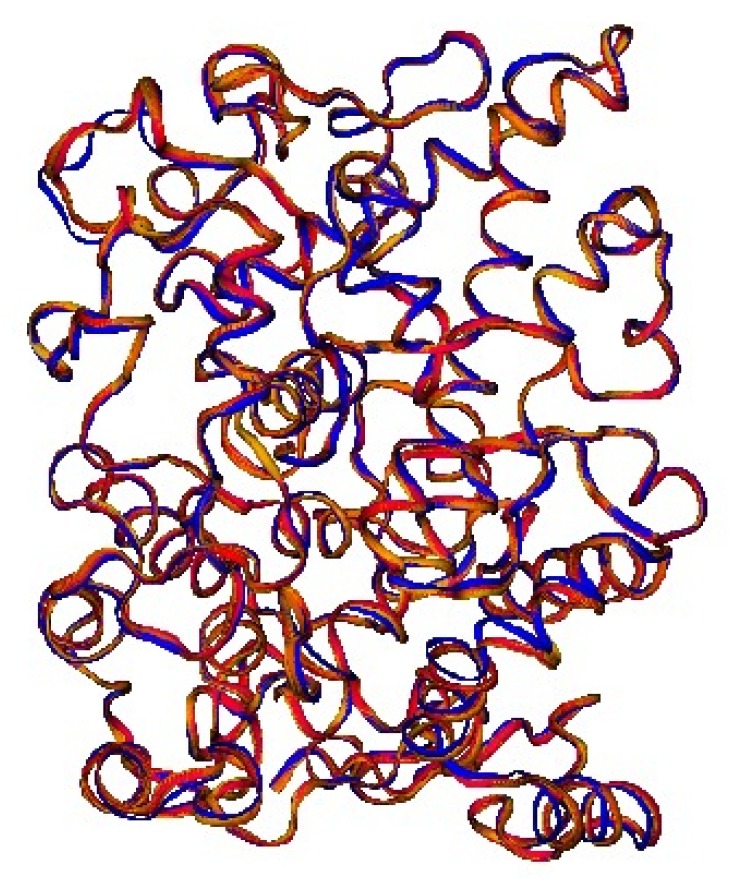
COX-1 structures. The figure reports the structures of PDB entries 1IGZ, 1IGX, 1FE2, 1U67 and 1DIY, that represent the cluster of structures with bound fatty acids described in the text (red and orange structures). The structure of the 1PGF (chain A) is reported in blue for comparison. All these structures belong to E.C 1.14.99.1, prostaglandin-endoperoxide synthase.

The random projection algorithm shows for this data set only a single cluster of structure, in which the structures that appeared in different cluster after PCA appear instead mixed. It should be noted that the differences between structures that are reported in different clusters by PCA are extremely small. In Figure 4 the superposition of the structures belonging to the cluster of structures with bound fatty acids appears almost perfect. In the same Figure 4 it is reported also a COX-1 structure that is very distant from those mentioned above [36]. As can be appreciated by inspecting the Figure, the differences are really minimal, so much so that it is not inconceivable that the PCA algorithm has operated a kind of over fitting of this data set. This observation is supported by the fact that the separation in different clusters vanishes in the two-dimensional random projection, suggesting also in this case that in reality only one cluster is present.

### 2.3. Hemoglobin: The Quintessence of Allostery

Hemoglobin (Hb) is undoubtedly the archetype of allosteric proteins [37,38]. Human adult hemoglobin (Hb A) has a tetrameric structure consisting of two α-chains and two β-chains with 141 and 146 amino acids respectively. Each of the chains in Hb contains a heme group, which is the binding site for ligands, such as oxygen, carbon monoxide, cyanide and nitric oxide. Hemoglobin usually drops in septic patients, due to a large number of factors, most of them still now undefined. The concentration of Hb in blood samples is currently accepted as a potent prognostic marker [39,40,41,42,43]. It is one of the first proteins whose structure was resolved by X-ray crystallography since the 1960s [44]. From these crystallographic data the Peruz’s two-structure and the Monod-Wyman-Changeux models for Hb allostery were proposed [45,46,47]. These classical models essentially postulates that the four subunits in Hb assume simultaneously either the tense (T) or relaxed (R) structures. Both structures can bind ligands but the affinity towards the ligands changes at the transition from the T to the R form. The differences in the observed crystal structures of the Hb in its oxy- and deoxy- forms are correlated with the T- and R- states of the Monod-Wyman-Changeux model.

Hb can be considered a dimer of αβ dimers. The two αβ dimers are in contact and assume a two-fold symmetry with the symmetry axis passing trough a water filled cavity composed by the four subunits. The helices B, G and H (the BGH frame) form a well packed structure that does not change upon ligand binding. The C and G helices and the FG corner of the unlike subunits make the sliding contacts that change upon oxygen binding. The classical results of Perutz suggested that upon oxygenation the $\alpha_{2}\beta_{2}$ dimer rotates relatively to the other dimer, the heme Fe(II) moves through the porphyrin plane and several several inter-subunit and intra-subunit salt bridges are broken. Actually dozen of different structures of Hb are available and the clustering and classification of these is still an active research field. Obviously we are not interested here in a systematic analysis of all these structure, but simply to a comparison between different methods of unsupervised clustering. However it should be mentioned that these systematic analyses have shown that what emerges is significantly more complicated than the simple two state model for the Hb structure [48].

We have included in the analysis only 30 Hb tetramer. The selection criterion was simply based on the search for the structure with the highest rank in the in the PDB cluster containing the α-chain of the human Hb A, using a 100% identity cutoff, and the constraint of exactly a tetramer presents in the structure and the absence of multiple coordinates for the same α-carbon atom in the pdb file. The structures are represented by a 30×1722 matrix.

**Figure 5:**
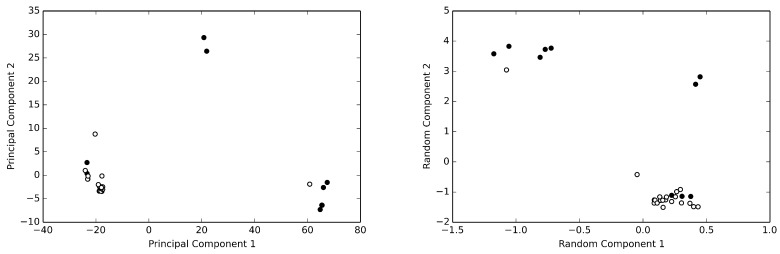
The human hemoglobin data set. Principal component analysis (**left panel**) and random projection analysis (**right panel**) are reported of the normalized data set containing the hemoglobin tetramers. Entries in this data set are reported as black circles if they represent liganded forms of the hemoglobin, or as withe circles if they are unliganded species. Two clusters of structures and two outliers are clearly detected by both algorithms. See text for further details.

The results of these analyses are reported in Figure 5. As can be appreciated, the Hb structures form two distinct groups in the two-dimensional projections, both in that obtained by means of the truncated SVD algorithm and in that one obtained from the random projection algorithm.

These two clusters correspond essentially to liganded and unliganded forms of the Hb, with few, and perfectly explicable, exceptions. The structures 1QSI, 1THB and 1YE2 [49,50,51], although representing liganded forms of the Hb molecule, cluster with the deoxy-Hb if analysed by both algorithms. However all the remaining structures represent T state of the molecule. Moreover 1SHR [52] clusters with the liganded forms of the Hb, despite being a deoxy-Hb, using both algorithms. Its particularity is justified considering that it is the structure of the Hb A${}_{2}$ with ferrocyanide bound. Interestingly, both algorithms report two structure as a separate mini-cluster, distinct both from the cluster containing the liganded structures and from that formed by the unliganded ones. These two structures (1SDK and 1SDL) have been obtained by using the trimesic acid for the avowed purpose of trapping the intermediates of the transition between the T form and the form R [53].

## 3. Discussion

In this work we have compared the effectiveness in dimensionality reduction for exploratory data analysis of two different algorithms. Both are capable to deal with degenerated data sets, i.e., data sets whose number of entries is much smaller than the number of the degrees of freedom that are required to describe the system. The first one is the truncated SVD method for the calculation of PCA [14], whereas the second one relies on random projection [18] which is based on the properties of random matrices [54,55,56,57] and the features of correlation matrices obtained from the protein dynamics [54,55,56,58,59].

The results of these analyses show that both algorithms are effective in the dimensionality reduction task, as well as the related cluster identification activity. If the same clusters are identified by means of the two algorithms, these can be considered valid. On the contrary, if clusters identified by the PCA are not observable using the method of random projections (in the same number of dimensions), a note of caution is required and the significance of the clusters must be evaluated in the light of biochemical knowledge about the protein. In this way, the technique of random projection represents a simple and intuitive way to evaluate the result of the PCA-based clustering algorithms.

We obtain the same cluster of structure by both algorithms in the case of HSA, COX-2 and Hb, with the single exception of the COX-1 case. However, as recalled above, it is a well known fact that a single stable structure is the dominant conformation of the COX-2, which is extremely similar to COX-1. Although it is true that there must be other conformations in the catalytic cycle of the COX-2, they must be only transient. This makes to think that, in the case of this enzyme, allostery models without conformational changes should be seriously taken into account. In fact plausible models of allostery without conformational changes have been proposed some time ago [60]. Our results suggest that this could be also the case for the COX-2 enzyme. The obtained data also show that the random projection can be a simple way to validate the data obtained by PCA in the presence of a number of data lower than the degrees of freedom of the system.

Sepsis induces changes in both protein synthesis and structure, independently from the general inflammatory response. The underlying inflammatory process takes place in order to neutralise the causative agents, also due to to various modifications of the metabolic asset and the generation of molecular isoforms of the biochemical pre- and newly formed mediators [61,62]. The availability of new tools for protein study may be perspectively useful to better understand such events and their possible implications for new diagnostic tests and more effective therapies.

## 4. Methods

Atomic coordinates of the selected proteins were obtained from PDB [2]. To obtain the data sets in a matrix form, the pdb files were loaded in VMD (Visual Molecular Dynamics) [63] and superposed by a simple Tcl (Tool command language) scrip (www.tcl.tk). The α-carbon atom coordinates were extracted from the updated pdb files and written in a text file such that each row described a structure by Tcl scripting. The raw text file were edited by Vim (Vi IMproved) scripting (www.vim.org), so as to obtain the data matrix in a readable file format by the numerical analysis software (see below).

When we are dealing with protein structure datasets, the correlation matrix (henceforth indicated as *C*) should be obtained from the Cartesian coordinates of the atoms included in the analysis that represent the degrees of freedom of the system (also covariance matrix could be used). In its classical implementation, the normalized PCA is based on the eigenvector decomposition of the correlation matrix [54,55,58,64,65,66]. After the centroid subtraction, the covariance matrix of the data set can be obtained as

$$\chi_{ij}=\langle \left(x_{i}-\langle x_{i}\rangle \right)\left(x_{j}-\langle x_{j}\rangle \right)\rangle$$

where 〈⋯〉 represents the average over all the conformations in the data set. Then the correlation matrix is calculated from the *C*-matrix as

$$P_{ij}=\frac{\chi_{ij}}{\sqrt{\chi_{ii}\chi_{jj}}}$$

and this square symmetric matrix is diagonalized as

$$R^{T}PR=\Lambda$$

using standard numerical routines, where *R* is an orthonormal transformation matrix, the superscript ${}^{T}$ means transposition and Λ is a diagonal matrix whose elements are the eigenvalues. The eigenvalues, and the corresponding eigenvectors, are ordered in descending order of the eigenvalues. The empirical matrix was projected onto the eigenvectors to give the so called principal components. To overcome the limitations imposed by the number of replicas required for the correct evaluation of the covariance matrix, algorithms have been proposed, able to estimate the principal components also in the case of not well dimensioned data sets [14,67]. However, PCA is not the only algorithm that can perform the dimensionality reduction and the related unsupervised clustering tasks. A new and promising class of unsupervised learning algorithms [18,68,69,70,71,72] is represented by those that use some random projection methods. Correlation matrices of the protein structures obtained from molecular dynamics experiments [54,55,56,58,59] exhibit spectra whose bulk eigenvalues can be modeled by some symmetric random matrices [54,55,56,57], suggesting that a random matrix [54,55,56,57] can be used to obtain a system on which to project the data set [18]. The random projection algorithm that will be used here [18] works exactly as PCA, with the only difference that the matrix *C* is replaced by a symmetric random matrix of the same dimension of *C*. This relax the minimum number of samples required for the analysis of data sets containing a large number of degrees of freedom, making then analyzable also small crystallographic data sets, in which the number of different structures is much smaller than the degrees of freedom required to describe a protein.

PCA and random projection algorithms were implemented numerically in the Python language (www.python.org) in an IPython notebook [73]. The NumPy numerical software library [74] was used, which is part of the Scipy [75] software package. Matplotlib [76] package was used to obtain the all graphical outputs (obtained from Scipy; www.scipy.org). Before proceeding with the analysis of data, a preprocessing step that can be described as

$$x_{std}^{i}=\frac{x^{i}-\mu_{x}}{\sigma_{x}}$$

was applied [66], where $\mu_{x}$ is the sample mean of a particular degree of freedom column and $\sigma_{x}$ the corresponding standard deviation, using the appropriate scikit-learn [77] built-in function. For PCA, the truncated SVD algorithm implemented in the scikit-learn software package was used [14,77]. The random projection algorithm and its practical implementation has been described in details elsewhere [18].

The confidence ellipses have been calculated assuming the normal distribution for the projected data and considering that the sum of the squares of Gaussian data is described by the Chi-square distribution.
